# Increased prevalence of clonal hematopoiesis of indeterminate potential in hospitalized patients with COVID-19

**DOI:** 10.3389/fimmu.2022.968778

**Published:** 2022-10-14

**Authors:** Judith Schenz, Katharina Rump, Benedikt Hermann Siegler, Inga Hemmerling, Tim Rahmel, Jan N. Thon, Hartmuth Nowak, Dania Fischer, Anna Hafner, Lucas Tichy, Katharina Bomans, Manja Meggendorfer, Björn Koos, Thilo von Groote, Alexander Zarbock, Mascha O. Fiedler, Johanna Zemva, Jan Larmann, Uta Merle, Michael Adamzik, Carsten Müller-Tidow, Torsten Haferlach, Florian Leuschner, Markus A. Weigand

**Affiliations:** ^1^ Department of Anesthesiology, Heidelberg University Hospital, Heidelberg, Germany; ^2^ Klinik für Anästhesiologie, Intensivmedizin und Schmerztherapie, Universitätsklinikum, Knappschaftskrankenhaus Bochum, Ruhr-University Bochum, Bochum, Germany; ^3^ CovidDataNet.NRW, Germany; ^4^ Department of Medicine, Cardiology, Heidelberg University Hospital, Heidelberg, Germany; ^5^ MLL Munich Leukemia Laboratory, Munich, Germany; ^6^ Department of Anesthesiology, Intensive Care and Pain Medicine, University Hospital Münster, Münster, Germany; ^7^ Department of Endocrinology, Diabetology, Metabolic Diseases and Clinical Chemistry, Heidelberg University Hospital, Heidelberg, Germany; ^8^ Department of Gastroenterology and Infectious Diseases, Heidelberg University Hospital, Heidelberg, Germany; ^9^ Department of Medicine, Hematology, Oncology and Rheumatology, Heidelberg University Hospital, Heidelberg, Germany; ^10^ University Center for ARDS and Weaning, Heidelberg University Hospital, Heidelberg, Germany

**Keywords:** cardiac function, CHIP, critically ill, immune system, SARS-CoV-2

## Abstract

Clonal hematopoiesis of indeterminate potential (CHIP) leads to higher mortality, carries a cardiovascular risk and alters inflammation. All three aspects harbor overlaps with the clinical manifestation of COVID-19. This study aimed to identify the impact of CHIP on COVID-19 pathophysiology. 90 hospitalized patients were analyzed for CHIP. In addition, their disease course and outcome were evaluated. With a prevalence of 37.8%, the frequency of a CHIP-driver mutation was significantly higher than the prevalence expected based on median age (17%). CHIP increases the risk of hospitalization in the course of the disease but has no age-independent impact on the outcome within the group of hospitalized patients. Especially in younger patients (45 – 65 years), CHIP was associated with persistent lymphopenia. In older patients (> 65 years), on the other hand, CHIP-positive patients developed neutrophilia in the long run. To what extent increased values of cardiac biomarkers are caused by CHIP independent of age could not be elaborated solely based on this study. In conclusion, our results indicate an increased susceptibility to a severe course of COVID-19 requiring hospitalization associated with CHIP. Secondly, they link it to a differentially regulated cellular immune response under the pressure of SARS-CoV-2 infection. Hence, a patient’s CHIP-status bears the potential to serve as biomarker for risk stratification and to early guide treatment of COVID-19 patients.

## Introduction

Clonal hematopoiesis of indeterminate potential (CHIP) is defined as the occurrence of an expanded proportion of mature blood cells derived from a single mutant hematopoietic precursor without evidence of hematological malignancies (1, 2). The principle behind the manifestation of CHIP is that the somatic mutation confers a certain predominance to the affected cell (3, 4). Different clinical consequences are linked to this expansion. Early reports have already described an association of clonal hematopoiesis with a higher mortality risk compared to individuals without CHIP-driver mutations (5, 6). Interestingly, this is not related to increased rates of cancer but associated in particular with increased cardiovascular mortality (7). Mechanistically, the development of clonal hematopoiesis is not only related to inflammatory processes, but it has even been identified as a driver of inflammation (8–12). In COVID-19, both inflammatory (13–15) and cardiac-associated processes (16–18) have been described. They are involved in the pathophysiology of the complex extra-pulmonary manifestations occurring in affected patients alongside pulmonary symptoms (19–21). Chronic infections with the human immunodeficiency virus and the thereby impacted inflammatory regulation have been linked already to an increased risk of age-related clonal hematopoiesis (22). Furthermore, it is known that the risk of numerous infections is increased by hematopoietic mosaic chromosomal alterations (23). Regarding CHIP and COVID-19 there is contradictory evidence. On the one hand, a stable CHIP prevalence and no outcome-relevant influence was described in a cohort including both hospitalized patients and outpatients with COVID-19 (24, 25). On the other hand, CHIP was reported being a risk factor for severe courses (26). With the aim of comprehensively assessing the impact of clonal hematopoiesis on the pathophysiology of COVID-19, hospitalized patients were evaluated for the presence of CHIP-driver mutations and an association between CHIP and disease progression.

## Materials and methods

### Study cohort

Patient recruitment was conducted at Heidelberg University Hospital, University Hospital Knappschaftskrankenhaus Ruhr University Bochum, and University Hospital Münster (all three: Germany). The ethics committees of the Medical Faculties of Heidelberg University (reference: S-176/2020), the Ruhr University Bochum (reference: 19-6606_6-BR), and the University of Münster (reference: SepsisDataNet.NRW: 2107-513-b-S, substantial amendment: CovidDataNet.NRW) approved this prospective observational clinical study. Patients were enrolled between October 2020 and August 2021. Written informed consent was obtained from all patients. If patients were incapable to give their consent, it was obtained from their legal representatives. All patients had a positive SARS-CoV-2 PCR test, were not vaccinated against SARS-CoV-2, and were admitted to the hospital due to COVID-19. Study inclusion was conducted within a maximum of 48 hours after initial admission to an intensive care unit (ICU) or general ward (time point: admission). Exclusion criteria were pregnancy, enrolment in an interventional study, preexisting immunosuppression, hematologic malignancies, or anemia.

Whole blood was drawn at enrolment. Upon centrifugation (2,000x*g*), the plasmatic fraction was removed and stored at -80°C for cytokine quantification. Peripheral blood mononuclear cells (PBMC) were separated directly by density gradient centrifugation and stored at -80°C until DNA extraction.

### DNA isolation

DNA isolation from PBMC was performed either with the DNeasy Blood & Tissue Kit (Qiagen, Hilden, Germany) or the Roche MagNA Pure System with the MagNAPure96 DNA and Viral NA LV Kit (Roche LifeScience, Mannheim, Germany).

### Sequencing, bioinformatics, and variant interpretation

Next-Generation Sequencing was performed for all samples by MLLSEQ - MLL Dx GmbH (Munich, Germany). The library preparation for enrichment was performed with 150ng DNA per sample with the Illumina TruSeq DNA Nano Kit (Illumina, San Diego, CA, USA) using Unique Dual Indices. Within the protocol, the DNA was fragmented to a length of 150bp using the Covaris LE220-plus ultrasonicator (Covaris, Woburn, MA, USA). Subsequently, the DNA target regions were enriched using the IDT Hybridization Capture Protocol and a corresponding custom lockdown gene panel (IDT Integrated DNA Technologies, Coralville, IA, USA). Sequencing of the libraries was performed on Illumina NovaSeq 6000 instruments (Illumina, San Diego, CA, USA) with paired end sequencing mode (2x101 cycles) and a target coverage of 4,000x.

The lockdown panel covered following genes: *ABL1, ASXL1, ATRX, BCOR, BCORL1, BRAF, CALR, CBL, CBLB, CBLC, CDKN2A, CEBPA, CSF3R, CUX1, DNMT3A, ETV6, EZH2, FBXW7, FLT3, GATA1, GATA2, GNAS, GNB1, HRAS, IDH1, IDH2, IKZF1, JAK2, JAK3, KDM6A, KIT, KRAS, KMT2A, MPL, MYD88, NOTCH1, NPM1, NRAS, PDGFRA, PHF6, PPM1D, PTEN, PTPN11, RAD21, RUNX1, SETBP1, SF3B1, SMC1A, SMC3, SRSF2, STAG2, TET2, TP53, U2AF1, WT1, ZRSR2*.

Ilumina’s BaseSpace Enrichment app (v3.1.1) was used to align the raw reads to hg19 reference sequence (Isaac Aligner v03.16.02.20). Subsequently, variants were called using PISCES (v5.1.3.60) somatic variant caller with 1% variant allele frequency (VAF) cutoff and 29 base quality filter and PCR duplicate flagging. In addition, the same data was processed through Illumina’s Dragen Enrichment app (v3.6.3) with 1% VAF, 1% VAF filter threshold and duplicate marking. We combined calls from both result files (VCF) for tertiary analysis.

The classification of the variants in mutated, variant of uncertain significance (VUS), or polymorphism was done using the public databases ClinVar, COSMIC, dbSNP, gnomAD, as well as the MLL in-house variant data base. Variants with a VAF <5% were validated in a subsequent amplicon-based assay.

### Flow cytometry

For quantification of HLA-DR expression on monocytes, 50µL freshly drawn whole blood was stained with 20µL BD Quantibrite anti-HLA-DR/anti-Monocyte PerCP-Cy5.5 reagent (clone: L243/MwP9) (BD Biosciences, Franklin Lakes, NJ, USA) for 30 minutes in the dark. For erythrocyte lysis, 450µL lysing solution (BD Biosciences, Franklin Lakes, NJ, USA) were added. Measurement was done immediately after incubation (15 minutes, in darkness). BD Quantibrite PE tubes (BD Biosciences, Franklin Lakes, NJ, USA) were used for quantifying the average number of HLA-DR molecules per monocyte as indicated by the manufacturer.

For quantification of lymphocyte subsets and monocytes, 50µL freshly drawn whole blood was stained with 20µL BD Multitest 6-color TBNK reagent and 5µL anti-Human CD14-V450 (clone: MφP9) using BD Trucount tubes to determine absolute counts (all BD Biosciences, Franklin Lakes, NJ, USA). After 30 minutes incubation in the dark, 450µL lysing solution (BD Biosciences, Franklin Lakes, NJ, USA) were added and measurement was performed immediately after 15 minutes incubation in darkness.

To identify T_reg_ cells and monocyte subsets, 100µL whole blood each were incubated (10 minutes) with 5μL Human TruStain FcX (BioLegend, San Diego, CA, USA) for Fc receptor blocking and stained by the addition of the appropriate antibodies (all from BD Biosciences, Franklin Lakes, NJ, USA) for 30 minutes at 4°C in the dark (T_reg_: 5µL anti-Human CD3-FITC (clone: UCHT1), 5µL anti-Human CD4-V500 (clone: RPA-T4), 20µL anti-Human CD25-PE (clone: M-A251), 20µL anti-Human CD127-Alexa Fluor 647 (clone: HIL-7R-M21); monocytes: 5µL anti-Human CD14-V450 [clone: MφP9), 5µL anti-Human CD16-FITC (clone: B73.1)]. Lysing of erythrocytes was done by adding 2mL lysing solution and incubation for 15 minutes at room temperature in darknes. Afterwards, the suspension was centrifuged (250x*g*, 5 minutes), the supernatant discarded, the cells washed (250x*g*, 5 minutes) once with 2mL CellWASH, and resuspended in FACSFlow (all from BD Biosciences, Franklin Lakes, NJ, USA).

A FACSLyric flow cytometer was used for all measurement. Results were analyzed using BD FACSuite software (both from BD Biosciences, Franklin Lakes, NJ, USA). Representative gating strategy is shown in **Supplementary Figure S1**.

### PBMC stimulation

1.5x10^5^ freshly isolated PBMC were resuspended in 300μL of RPMI 1640 (Thermo Fisher Scientific Inc, Waltham, MA, USA), containing GlutaMAX, 100units/mL penicillin, 100μg/mL streptomycin and 10% heat-inactivated fetal bovine serum ultra-low endotoxin (Cell Concepts, Umkirch, Germany). Stimulation was performed with 3μL Dynabeads Human T-Activator CD3/CD28 (Thermo Fisher Scientific, Waltham, MA, USA). Prior to use, Dynabeads were washed and resuspended in culture medium according to the manufacturer’s instruction. Control cells were incubated without a stimulating agent. Following a 24h incubation (37°C, 5% CO_2_), supernatants were collected by centrifugation (1,000x*g*, 5 minutes) and stored at -80°C until cytokine quantification.

### Cytokine quantification

Cytokine levels in plasma or supernatants were measured using colorimetric enzyme-linked immunosorbent assay (ELISA) according to the respective manufacturer’s instructions. For CXCL10/IP-10 (limit of detection (LOD): 31.2pg/mL), TGF-β1 (LOD: 31.2pg/mL), and IL-6 (LOD: 9.4pg/mL) the respective human Duo-Set ELISA (all from R&D Systems, Minneapolis, MN, USA) were used. IFN-γ (LOD: 0.06pg/mL) was measured using the IFN gamma Human ELISA Kit, High Sensitivity (Invitrogen, Thermo Fisher Scientific Inc, Waltham, MA, USA).

### Statistical analyses

All statistical analyses were performed using SPSS Statistics (Version 27.0.1.0, IBM^®^, Armonk, NY, USA). Continuous variables are presented as median (interquartile range (IQR)). Two-sided Mann–Whitney U-test or Kruskal-Wallis test were used for comparison, as appropriate. Categorical variables are shown as absolute number (frequency) and compared using Chi-square test or Fisher’s exact test, as appropriate. Binomial test was used to compare an observed prevalence against an expected. Figures were created using GraphPad Prism (V9.3.1 for Windows, GraphPad software, La Jolla, CA, USA).

## Results

### Patient characteristics

In total, 90 patients hospitalized due to COVID-19 were included in the study (**Table 1**). 72 of these patients were critically ill and treated in an ICU at the time of inclusion. The other 18 patients were admitted to general wards. Two of them were transferred to an ICU at a later stage. Patients had a median age of 60.5 (52.0 – 69.3) years and were overweight (body mass index: 28.4 (24.4 – 32.6) kg/m^2^). 35.6% of the cohort were female. At admission, only 35.6% of the patients were spontaneously breathing and without supplemental oxygen demand or respiratory support.

**Table 1:** Baseline characteristics.

	Total cohort (n=90)	CHIP-positive (n=34)	CHIP-negative (n=56)	P-value
Age, years				0.003
Median (IQR)	60.5 (52.0 – 69.3)	66.5 (56.8 – 74.8)	55.5 (50.3 – 65.8)	
Range	24 – 95	45 – 95	24 – 87	
Sex, no. (%)				1.000
Female	32 (35.6%)	12 (35.3%)	20 (35.7%)	
Male	58 (64.4%)	22 (64.7%)	36 (64.3%)	
Body mass index, kg/m^2^				0.188
Median (IQR)	28.4 (24.4 – 32.8)	29.7 (25.7 – 34.6)	28.3 (24.1 – 31.5)	
Range	17.3 – 45.2	23.4 – 45.2	17.3 – 39.9	
	n=48	n=14	n=34	
SOFA score at ICU admission				0.311
Media (IQR)	4.0 (2.0 – 10.0)	5.0 (3.0 – 10.0)	4.0 (2.0 – 9.8)	
Range	0 – 17	0 – 17	0 – 16	
Hospitalization, days				0.962
Media (IQR)	18.5 (10.8 – 39.0)	20.5 (11.8 – 34.0)	18.0 (10.0 – 40.8)	
Range	3 – 84	3 – 61	3 – 84	
Length of ICU stay, days				0.582
Media (IQR)	10.0 (3.0 – 27.0)	10.0 (3.8 – 21.0)	10.5 (3.0 – 30.8)	
Range	0 – 83	0 – 39	0 – 83	
Discharge status, no. (%)				0.145
Deceased	24 (26.7%)	13 (38.2%)	11 (19.6%)	
Home	46 (51.1%)	16 (47.1%)	30 (53.6%)	
Rehabilitation clinic	5 (5.6.%)	1 (2.9%)	4 (7.1%)	
Transfer to peripheral hospital	14 (15.6%)	3 (8.2%)	11 (19.3%)	
Other	1 (1.1%)	1 (2.9%)	0 (0.0%)	
Ventilation at admission, no. (%)				0.926
Extracorporeal membrane oxygenation	3 (3.3%)	1 (2.9%)	2 (3.3%)	
Invasive ventilation	17 (18.9%)	5 (14.7%)	12 (21.4%)	
Non-invasive ventilation	21 (23.3%)	9 (26.5%)	12 (21.4%)	
Nasal high flow therapy	17 (18.9%)	6 (17.6%)	11 (19.6%)	
Spontaneously breathing	32 (35.6%)	13 (38.2%)	19 (33.9%)	

P-values from two-sided Fisher’s exact test for sex, Chi-square test for discharge status and ventilation at admission, and from two-sided Mann–Whitney U-test for comparison of continuous variables.

### Prevalence of CHIP-associated mutations

**Figure 1:**
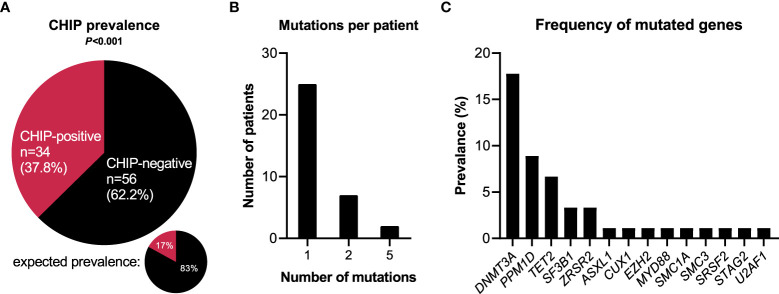
CHIP-associated mutations in hospitalized patients with COVID-19. **(A)** Prevalence of clonal hematopoiesis in hospitalized patients with COVID-19. CHIP-positive = patients carrying at least one CHIP-driver mutation with a VAF≥0.01. Expected prevalence according to Watson et al. (4). Observed and expected prevalence were compared using binomial test. Statistically significant results (P ≤ 0.05) are highlighted by bold print. **(B)** Number of individual mutations per CHIP-positive patient. **(C)** Prevalence broken down by affected genes.

With a VAF ≥ 1%, at least one CHIP-driver mutation was detected in 34 out of the 90 patients included in the study, corresponding to a prevalence of 37.8% (**Figure 1A**). According to modeling by Watson et al. including ~50,000 unselected individuals, 1% VAF cutoff at the median age of our cohort would be expected to result in a prevalence of single-mutant clones of 17% (*P*<0.001) (4). Thus, the frequency of a CHIP-driver mutation is significantly higher in hospitalized COVID-19 patients than in the general population adjusted for age.

Among the 34 CHIP-positive patients, 25 patients (73.5%) showed one single gene mutation, seven patients (20.6%) had two mutations, and two cases (5.9%) showed five mutations (**Figure 1B**). Most frequently mutated genes were *DNMT3A* (16/90 = 17.8%), *PPM1D* (8/90 = 8.9%), *TET2* (6/90 = 6.7%), *SF3B1* (3/90 = 3.3%), and *ZRSR2* (3/90 = 3.3%) (**Figure 1C**). Of the 49 single mutations identified, 32 (65.3%) were single nucleotide variants, nine (18.4%) were deletion mutants, seven (14.3%) were duplication mutants, and one (2.0%) was an insertion mutant (**Supplementary Table S1**).

### Prognostic relevance of CHIP-driver mutations for disease progression

**Figure 2:**
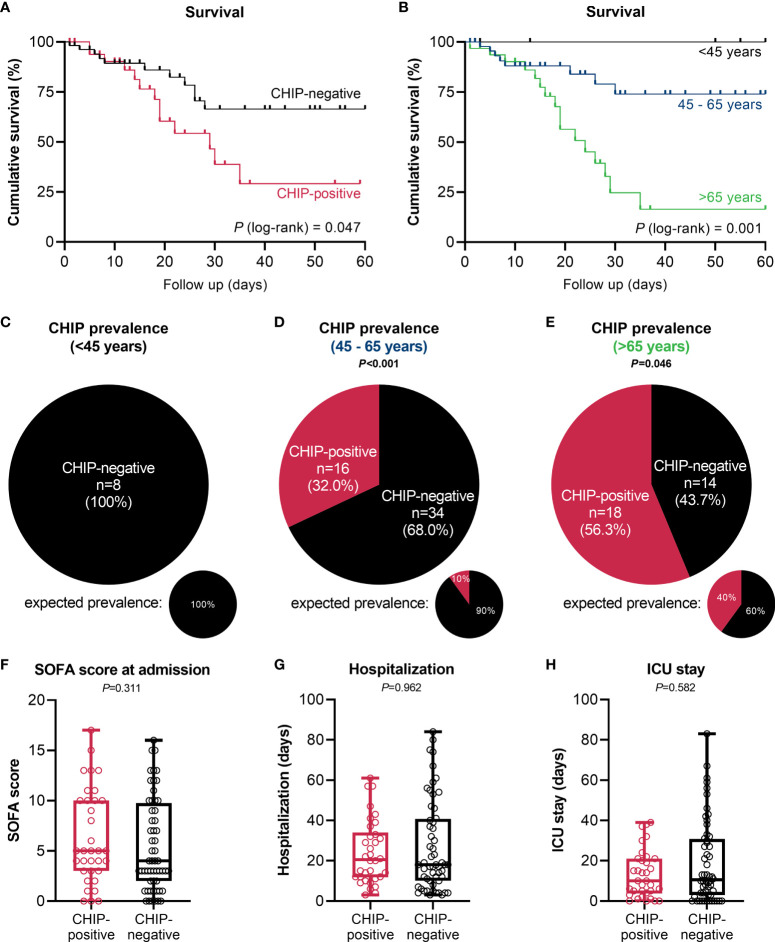
Impact of clonal hematopoiesis on patient outcome. **(A)** Kaplan-Meier survival curve for overall survival of CHIP-positive (n=34) vs. CHIP-negative (n=56) patients within a 60-day period. **(B)** Kaplan-Meier survival curve for overall survival of patients <45 years (n=8), 45 – 65 years (n=50), and >65 years (n=32) within a 60-day follow-up period. Statistical evaluations were done using log-rank Mantel-Cox test. CHIP prevalence in patients **(C)** <45 years, **(D)** 45 – 65 years, and **(E)** >65 years. Expected prevalences according to Watson et al. (4). Observed and expected prevalence were compared using binomial test. **(F)** SOFA score at admission, and length of **(G)** hospitalization and **(H)** ICU stay. Each data point represents an individual patient (CHIP-positive n=34; CHIP-negative n=56). Horizontal line within the box marks the median, boxes depict the IQR, and whiskers indicate the total range. Group comparisons were performed by two sided Mann–Whitney U-test. Statistically significant results (P ≤ 0.05) are highlighted by bold print.

The occurrence of at least one CHIP-driver mutation was associated with a significantly inferior clinical outcome in terms of survival within a 60-day follow-up period (**Figure 2A**). Since both groups significantly differed for age (CHIP-positive: 66.5 years (56.8 – 74.8); CHIP-negative: 55.5 years (50.3 – 65.8); *P*=0.003) (**Table 1**), Cox proportional regression analysis was performed to account for the potential effect of age: Only age was independently associated with survival, whereas carrying a CHIP-driver mutation was not (**Table 2**). Therefore, three subgroups were defined according to age (<45 years: n=8, 45-65 years: n=50, >65 years: n=32). Again, with these subgroups, it was confirmed that older patients had a significantly worse survival (**Figure 2B**). It is therefore not surprising that within these subgroups there is no difference in survival between patients with and without CHIP-driver mutation (**Supplementary Figure S2A**). There were no differences in age between CHIP-positive and -negative patients, within the age groups (**Supplementary Figure S2B**).

**Table 2:** Cox proportional regression with age and CHIP status included as covariates.

	HR	95% CI	P-value
Overall survival			
Age	1.093	1.052 – 1.135	<0.001
CHIP status (CHIP-positive vs. CHIP-negative)	1.254	0.547 – 2.877	0.593

In the group of patients younger than 45 years (median: 36.0 (26.3 – 42.5) years), no CHIP-driver mutation was detected (**Figure 2C**). In patients aged 45 to 65 years (median: 55.0 (52.0 – 61.3) years), 32% harbored a mutation (**Figure 2D**). According to Watson et al. (4), the expected prevalence at the subgroup’s median age would be only 10% (*P*<0.001). A similar result was revealed for patients older than 65 years (**Figure 2E**). With a median age of 75.0 (68.3 – 80.8) years in this subgroup, 40% prevalence would be expected according to the modeling. In contrast, among the hospitalized COVID-19 patients the prevalence was 56.3% (*P*=0.046).

Beyond age, the groups of CHIP-positive and -negative patients notably did not differ in terms of sex (**Table 1**), SOFA score at admission (**Figure 2F**), length of hospitalization and ICU stay (**Figures 2G, H**), or concerning other baseline characteristics (**Table 1**). This is also true when comparing the two groups based on age (**Supplementary Figures S3A, B**) or comparing the three age groups with each other (**Supplementary Figure S3C**).

### Association between clonal hematopoiesis and organ function

**Figure 3:**
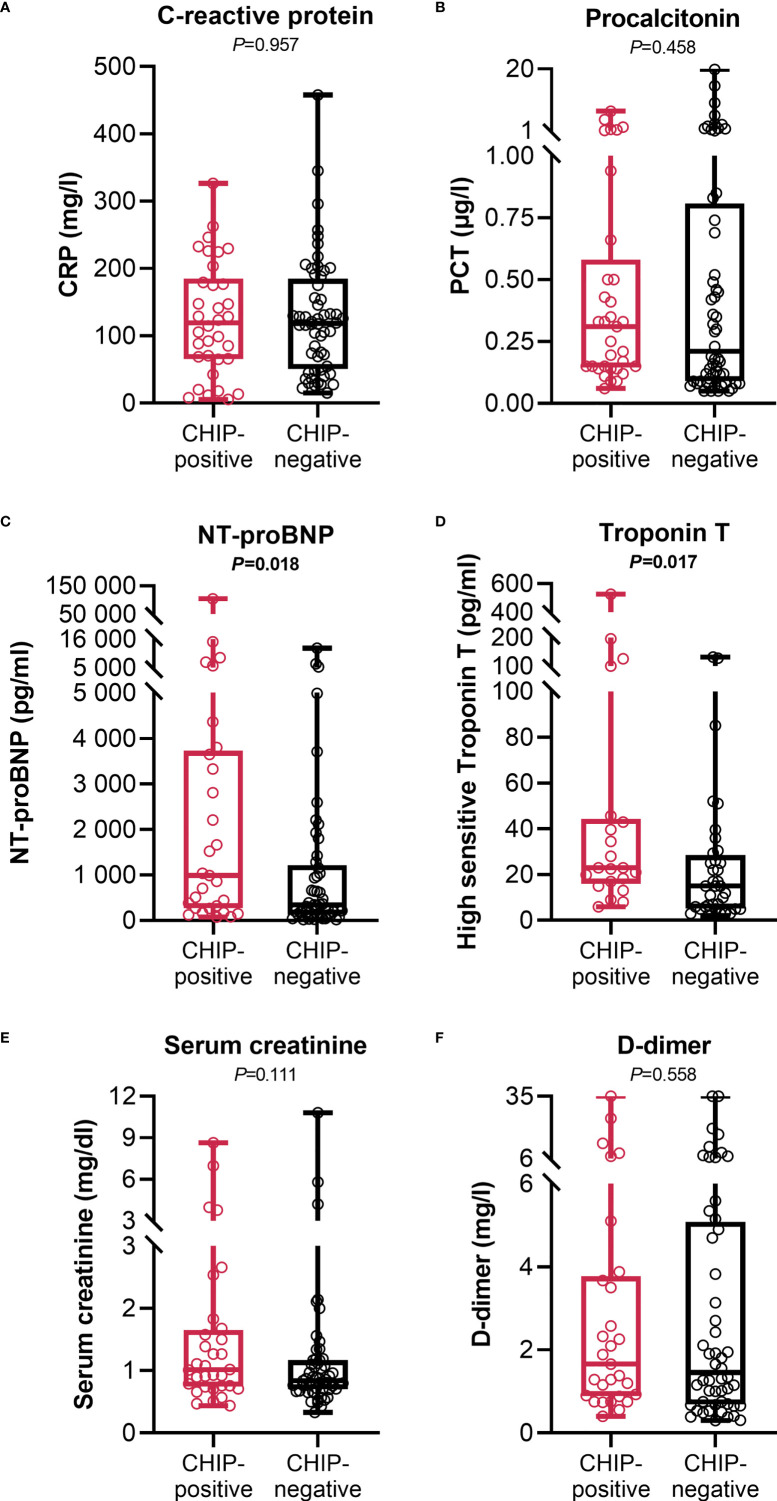
Infection and organ function related clinical parameters at admission. **(A)** C-reactive protein (CHIP-positive: n=34; CHIP-negative: n=56), **(B)** procalcitonin (CHIP-positive: n=33; CHIP-negative: n=56), **(C)** NT-proBNP (CHIP-positive: n=29; CHIP-negative: n=49), **(D)** high sensitive Troponin T (CHIP-positive: n=21; CHIP-negative: n=36), **(E)** serum creatinine (CHIP-positive: n=32; CHIP-negative: n=55), and **(F)** D-dimer (CHIP-positive: n=29; CHIP-negative: n=52) levels at admission. Each data point represents an individual patient. Horizontal line within the box marks the median, boxes depict the IQR, and whiskers indicate the total range. Group comparisons were performed by two sided Mann–Whitney U-test. Statistically significant results (P ≤ 0.05) are highlighted by bold print.

**Figure 4:**
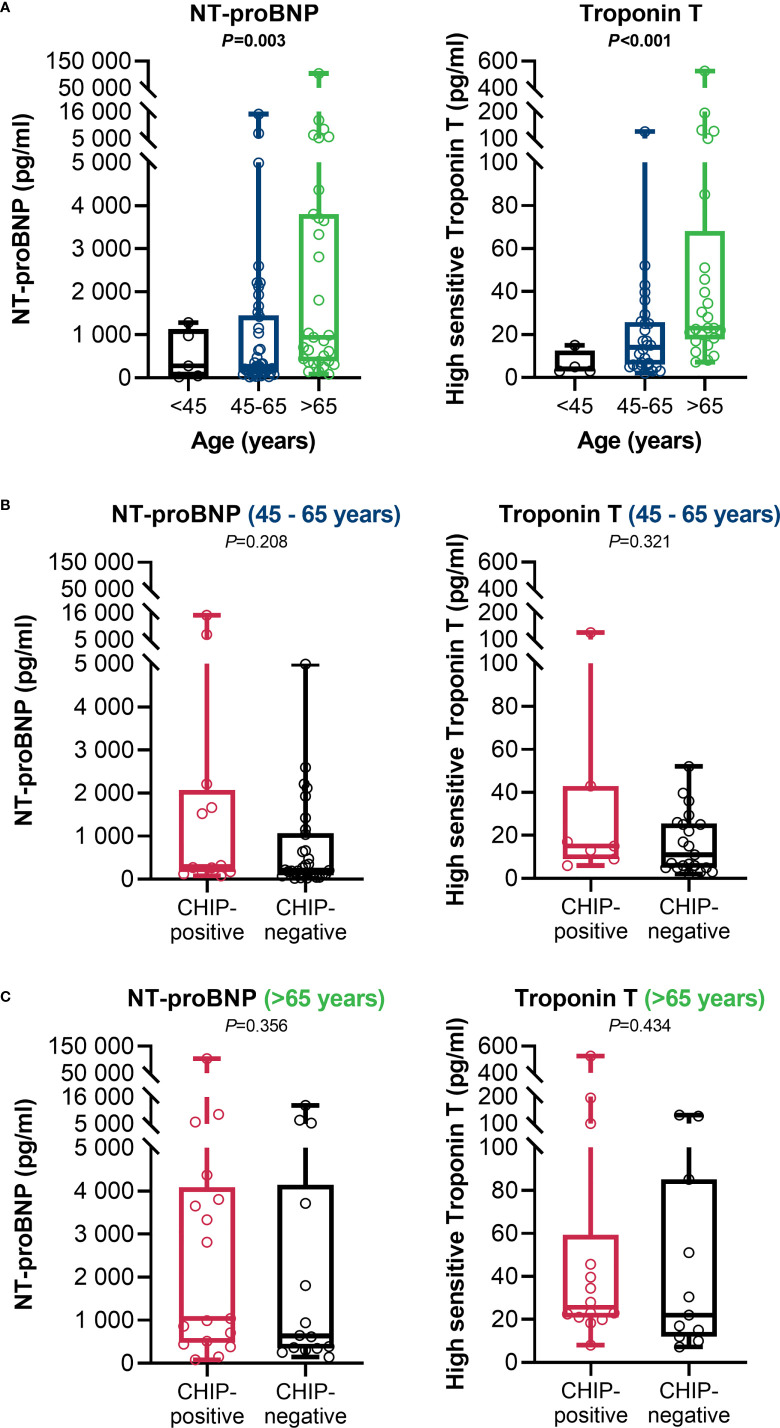
Age-adjusted cardiac function related clinical parameters at admission. **(A)** NT-proBNP (< 45: n=5; 45 – 65: n=42; > 65: n=31) and high sensitive Troponin T (<45: n=4; 45 – 65: n=28; >65: n=25) in comparison between the age groups. Group comparisons were done using Kruskal-Wallis test. **(B)** NT-proBNP (CHIP-positive: n=12; CHIP-negative: n=30) and Troponin T (CHIP-positive: n=7; CHIP-negative: n=21) in the 45 – 65 age group. **(C)** NT-proBNP (CHIP-positive: n=17; CHIP-negative: n=14) and Troponin T (CHIP-positive: n=14; CHIP-negative: n=11) for the > 65-year-olds. Group comparisons were performed by two sided Mann–Whitney U-test. Each data point represents an individual patient. Horizontal line within the box marks the median, boxes depict the IQR, and whiskers indicate the total range. Statistically significant results (P ≤ 0.05) are highlighted by bold print.

At admission, CHIP-positive and -negative patients had comparable supplemental oxygen demand and required invasive or non-invasive ventilation to a comparable extent (**Table 1**). Besides, there were no differences regarding Horovitz index (**Supplementary Table S2**). Therefore, a similar degree of lung injury can be assumed in both groups. The infection-related parameters C-reactive protein (**Figure 3A**) and procalcitonin (**Figure 3B**) likewise did not vary between CHIP-positive and CHIP-negative patients. Surprisingly, investigating biomarkers for organ functions (**Supplementary Table S2**), at admission, the two groups differed only, but significantly regarding the cardiac biomarkers NT-proBNP (**Figure 3C**) and Troponin T (**Figure 3D**). Both markers already showed a clinically relevant increase in the CHIP-negative group (NT-proBNP: 347.0 (133.8 – 1217.0) pg/ml; Troponin T: 15.0 (5. – 28.5) pg/ml). Yet, the increase was markedly higher in the group carrying a CHIP-driver mutation (NT-proBNP: 991.9 (270.5 – 3726.0) pg/ml; Troponin T: 23.0 (16.0 – 44.3) pg/ml). High levels of cardiac markers were not related to poorer survival (**Supplementary Figure S4**). However, when both NT-proBNP and Troponin T were compared in relation to age, significantly higher values were again found in older patients (**Figure 4A**). Within the age groups, no differences were found between CHIP-positive and -negative patients regarding these cardiac markers, neither for those aged 45 – 65 years (**Figure 4B**) nor for those over 65 years (**Figure 4C**). Normal values for serum creatinine were found in patients with and without a CHIP-driver mutation (**Figure 3E**). D-dimer levels did not differ between both groups (**Figure 3F**).

### Association between clonal hematopoiesis and peripheral immune parameters

At admission, immune parameters were comprehensively determined from whole blood from a 42-patient subcohort to provide evidence of the mechanistic link between clonal hematopoiesis, immune function, and poorer patient outcome (**Supplementary Table S3**). No differences were found between CHIP-positive and CHIP-negative patients concerning their absolute numbers of different lymphocytic populations and monocyte subpopulations or HLA-DR expression on monocytes. Plasma cytokine levels for IFN-γ, IP-10, TGF-β, and IL-6 also did not differ. Moreover, the cytokine response to *ex vivo* stimulation (αCD3/αCD28) of freshly isolated PBMC was found to be comparable.

**Figure 5:**
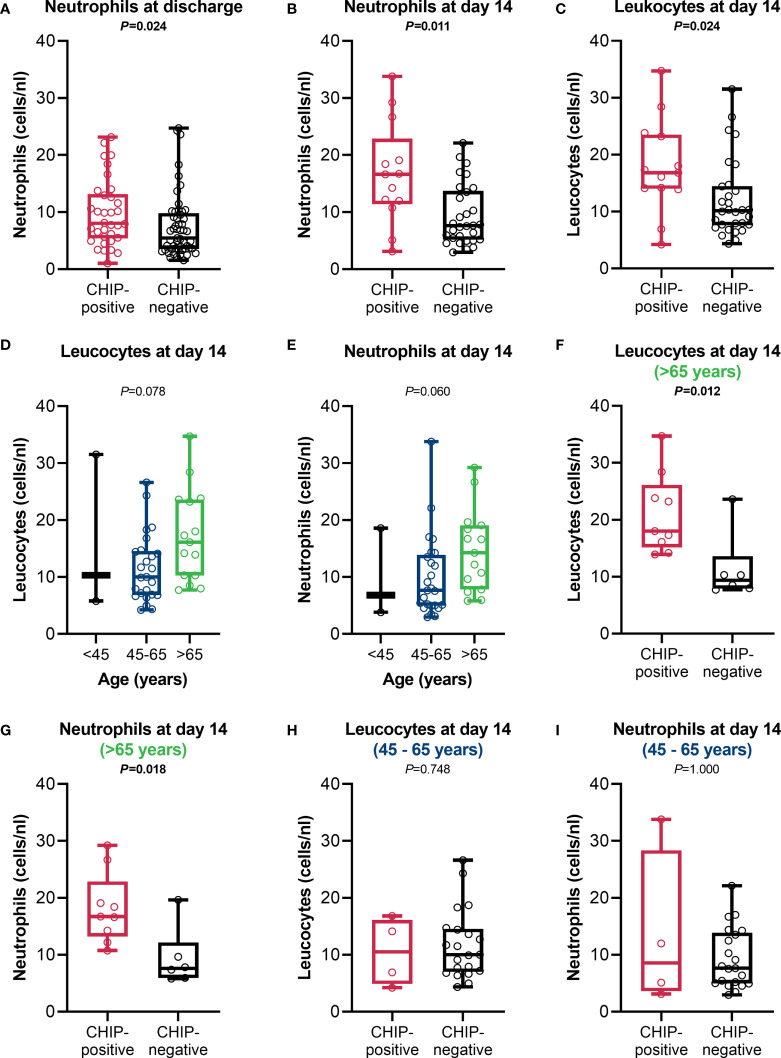
Leucocyte and neutrophil counts. Neutrophil counts at **(A)** discharge (CHIP-positive: n=34; CHIP-negative: n=53), **(B)** day 14 after admission, and **(C)** leucocyte counts at day 14 after admission (CHIP-positive: n=13; CHIP-negative: n=30). **(D)** Leucocyte and **(E)** neutrophil counts in comparison between the different age groups at day 14 after admission (<45: n=3; 45-65: n=25; >65: n=15).**(F)** Leucocyte and **(G)** neutrophil counts for patients aged >65 years (CHIP-positive: n=9; CHIP-negative: n=6) and **(H, I)** 45 – 65 years (CHIP-positive: n=4; CHIP-negative: n=21) at day 14 after admission. Group comparisons were performed by two sided **(A–C, F–I)** Mann–Whitney U-test or **(D, E)** Kruskal-Wallis test. Each data point represents an individual patient. Horizontal line within the box marks the median, boxes depict the IQR, and whiskers indicate the total range. Statistically significant results (P ≤ 0.05) are highlighted by bold print.

Since the immune parameters at admission did not differ between CHIP-positive and CHIP-negative patients, parameters of the differential blood count routinely determined during treatment were used for comparison over time from the entire cohort (**Supplementary Table S4**). As expected, there were no differences between the groups at admission. However, at discharge, differences were found for lymphocytes, neutrophils, and eosinophils. Patients with a CHIP-driver mutation had higher neutrophil counts than patients without such a mutation (8.0 (5.4 – 13.2) vs. 5.5 (3.6 – 9.8) cells/nL; *P*=0.024)) (**Figure 5A**). These values indicate that especially CHIP-positive patients show neutrophilia (>7.7 cells/nL). Since the timepoint “discharge” has a very variable time lag to the timepoint “admission” depending on the course of the disease, these parameters were also compared at day 7 [total cohort: 64/90 (71.1%), CHIP-positive: 24/34 (70.6%), CHIP-negative: 40/56 (71.4%)] and day 14 (total cohort: 43/90 (47.8%), CHIP-positive: 13/34 (38.2%), CHIP-negative: 30/56 (53.6%)) after admission, including all patients who were still treated in the original hospital at that time. At day 7, both groups showed no significant alterations (**Supplementary Table S4**). The difference in neutrophil counts only became apparent at day 14 after admission (**Figure 5B**). The still more pronounced neutrophilia at this timepoint compared to “discharge” seems to be causative for the leukocytosis occurring in patients with mutation (**Figure 5C**). Albeit the group over 65 years of age tended to have higher leukocyte (**Figure 5D**) and neutrophil (**Figure 5E**) counts compared to the younger patients, the significant difference between CHIP-positive and -negative patients was maintained in this age group (**Figures 5F, G**). Among those aged 45 – 65 years, however, no difference was found (**Figures 5H, I**).

**Figure 6:**
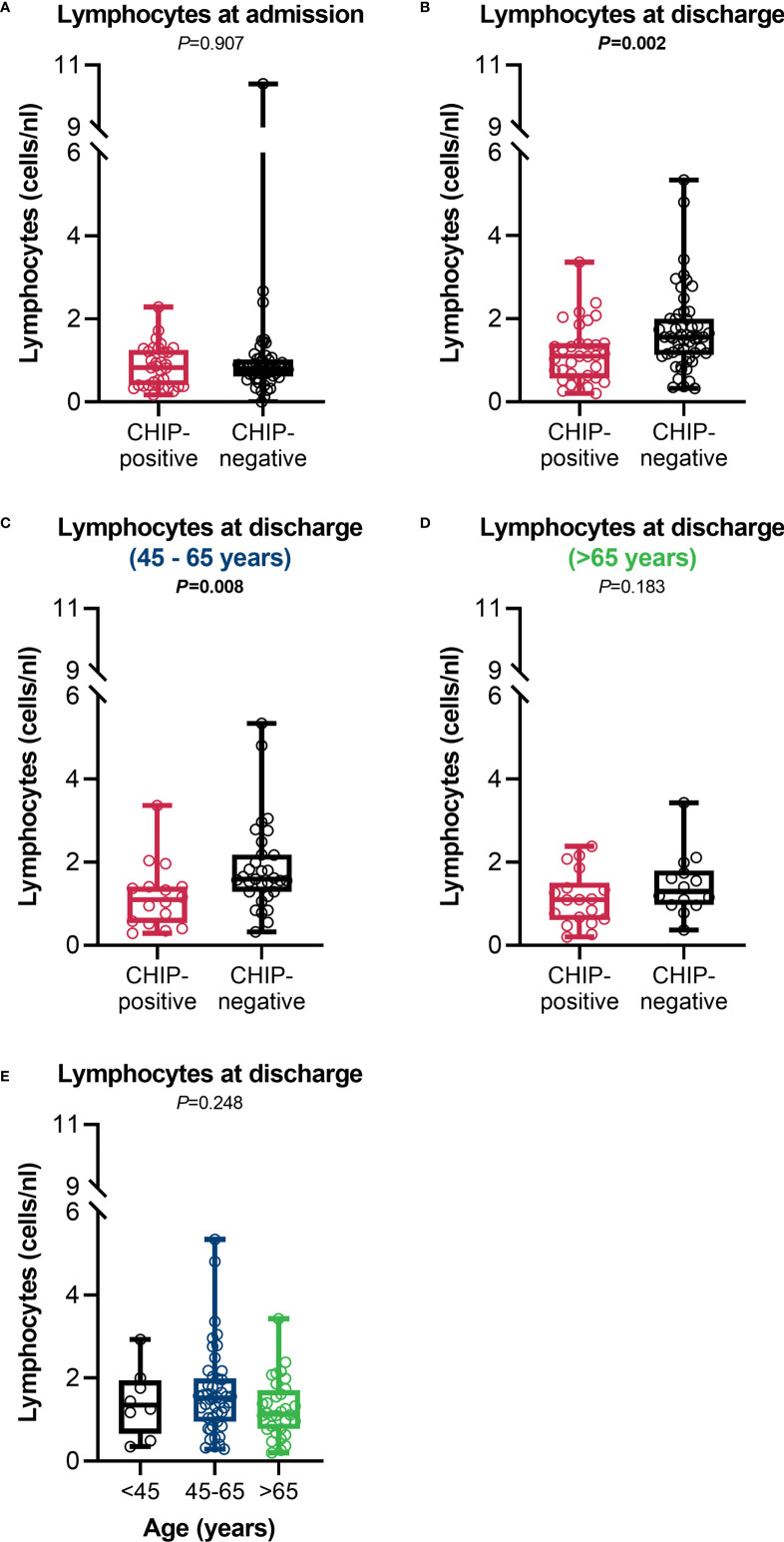
Lymphocyte counts. Lymphocyte counts at **(A)** admission (CHIP-positive: n=34; CHIP-negative: n=53) and **(B)** discharge (CHIP-positive: n=34; CHIP-negative: n=53). Lymphocyte counts at discharge for patients aged **(C)** 45 – 65 years (CHIP-positive: n=16; CHIP-negative: n=31) and **(D)** > 65 years (CHIP-positive: n=18; CHIP-negative: n=14). **(E)** Lymphocyte counts at discharge in comparison between the different age groups (<45: n=8; 45-65: n=47; >65: n=32). Group comparisons were performed by two sided **(A–D)** Mann–Whitney U-test or **(E)** Kruskal-Wallis test. Each data point represents an individual patient. Horizontal line within the box marks the median, boxes depict the IQR, and whiskers indicate the total range. Statistically significant results (P ≤ 0.05) are highlighted by bold print.

While both groups were lymphopenic (<1.0 cells/nL) at admission (0.8 (0.4 – 1.3) vs. 0.8 (0.6 – 1.0); *P*=0.907) (**Figure 6A**), patients with a CHIP-driver mutation recovered worse and had significantly lower lymphocyte counts at discharge than patients without a mutation (1.1 (0.6 – 1.4) vs. 1.6 (1.1 – 2.0); *P*=0.002) (**Figure 6B**). Here again, differences between the age groups were apparent. Especially in the group of 45 – 65-year-olds, CHIP-positive patients poorly overcame lymphopenia and had significantly lower lymphocyte counts at discharge compared to patients without clonal hematopoiesis (**Figure 6C**). CHIP-negative patients older than 65 years were less efficient in controlling their lymphopenia, thus presenting with similarly low lymphocyte levels as CHIP-positive patients (**Figure 6D**). When comparing the age groups with each other, the lymphocyte counts were comparable (**Figure 6E**). Surprisingly, patients without a mutation had higher eosinophil granulocyte counts but without clinical relevance (eosinophilia >0.5 cells/nL) (**Supplementary Table S4**). Although not statistically significant, a trend in both lymphocytes and eosinophils might also be seen when comparing patients at day 14 (**Supplementary Table S4**).

## Discussion

Investigating the impact of CHIP in patients with COVID-19, we demonstrate here that CHIP prevalence in patients hospitalized due to COVID-19 is significantly higher than in the general population. Hence, and in line with Bolton et al. (26), CHIP increases the risk of a severe course of the disease. This also holds true when subgroups are evaluated separately according to age. Remarkably, Petzer et al. reported lower prevalences and no link with severe courses (24). This dichotomy might be explained by a higher VAF cutoff of 2% compared to 1% in our study. Intriguingly, based on our data, we reveal that CHIP leads to a differentially regulated cellular immune response under the pressure of SARS-CoV-2 infection. This becomes apparent with age-dependent differences especially in neutrophils and lymphocytes only in the later course. Furthermore, the presence of a respective driver mutation seems to be mechanistically related to the COVID-19 risk factor age. Thus, the results of our study provide novel insights into the group of nonmalignant diseases in which clonal hematopoiesis is closely linked to adverse courses or outcome.

Within our cohort, the group harboring a CHIP-driver mutation was significantly older than the group without a corresponding mutation. This is consistent with previous studies analyzing patients hospitalized for COVID-19 (25, 27) and with the fact that clonal hematopoiesis is an age-associated process (7). However, for COVID-19, age itself is a well-known risk factor for mortality (28, 29) and only age remained independently associated with survival in our cohort. Thus, for patients hospitalized for COVID-19, CHIP alone does not impact survival. The markedly higher prevalence of CHIP in these patients compared with an overall population, however, was still observed within the subgroups formed according to age. CHIP is, therefore, a risk factor for COVID-19 courses requiring hospitalization but does not directly affect outcome within the hospitalized group. The cohort unintentionally includes mainly overweight patients. Obesity is a well-described risk factor for COVID-19-related hospitalization (30). Against this background, it is to be expected that a large proportion of the cohort is overweight. However, since CHIP-positive and -negative patients did not differ with respect to their body mass index, this factor can be excluded as an influencing factor in our study. However, it should be mentioned that a possible link between obesity and CHIP is the subject of current investigations. Especially in the context of cardiovascular diseases, which correlates with both CHIP and obesity, a direct mechanistic link between CHIP and obesity is discussed (31, 32). In addition, our study did not analyze other risk factors or preexisting conditions associated with a high risk of COVID-19-related hospitalization.

CHIP-positive and -negative patients had a comparable disease severity at the time of admission to ICU or general ward, respectively, underscoring the role of CHIP as a risk factor for severe courses. Particularly their SOFA scores, as an established measure to predict clinical outcome at an early stage, especially in critically ill patients, did not differ. Severity of SARS-CoV-2-induced lung injury was also comparable in both groups. Moreover, both groups showed no differences in further infection- or organ function-related biomarkers, with exception of the cardiac marker NT-proBNP and Troponin T. The increase in both parameters was significantly greater in the CHIP-positive patients. Cardiac involvement in the pathophysiological appearance of severe COVID-19 has been described in detail previously (33, 34) and might be the underlying reason for the comparatively slight increase in CHIP-negative patients compared to standard values. Concurrent with the effect on survival, the observed difference equalizes in age-adjusted subgroups. Several studies revealed CHIP bearing an excessive cardiovascular risk and linked it, among others, to atherosclerosis, coronary artery diseases, or degenerative aortic valve stenosis (35–37). Based on our data, we cannot distinguish whether the tremendous increase in the CHIP-positive cohort can be related to clonal hematopoiesis or is simply a consequence of cardiac risk increasing with age. The interrelation and mutually reinforcing effect of all these factors, nevertheless, is unequivocal. Furthermore, it is in line with a modeling-based proposition formulated in June 2020 that COVID-19 mortality is linear correlated with CHIP frequency (38). Further investigations are needed to provide evidence if a CHIP-related higher, possibly previously unknown cardiac burden leads to deteriorated courses of COVID-19 or if CHIP fuels cardiac involvement in the manifestations of COVID-19.

Mas-Peiro et al. recently detected higher levels of proinflammatory subsets of circulating T cells and monocytes in patients with degenerative aortic valve stenosis carrying DNMT3A- or TET2-CHIP mutations (39). Avagyan et al. provided evidence, that clonal fitness is related to upregulation of anti-inflammatory signaling pathways in the mutant progenitor cells, leading to resistance to inflammatory signaling in their mature cellular progeny (40). Hence, it can be assumed that alongside with changes in cardiac function, immune response related differences are contributing to the higher disease severity in CHIP-positive patients. To our surprise, this only became detectable in the prolonged clinical course. At the time of admission, only lymphopenia, typical of severe COVID-19 (41, 42), as well as a weak T-cell response to *ex vivo* stimulation were apparent in both CHIP-positive and -negative patients, while there were no differences related to the immune response between the two groups. Patients without CHIP-driver mutations were able to overcome the lymphopenia, whereas in patients with such a mutation, long lasting neutrophilia may indicate persistent inflammation. Remarkably, these two, immune system-related, effects of CHIP do not appear to be necessarily linked but rather to vary in strength age-dependently. Protracted lymphopenia is most evident in the younger (45 – 65-years-old) patients. The contrast between CHIP-positive and CHIP-negative patients is hardly noticeable in the older ones, as here the CHIP-negative ones recover less well. However, there is persistent neutrophilia in this age group of over 65 years, which is not even a trend in the younger patients.

Taken together, our study substantiates the findings that harboring acquired somatic mutations in hematopoietic cells linked with CHIP amplifies the risk for hospitalization over the course of COVID-19. Additionally, we provide evidence that CHIP leads to distinct, late-occurring alterations of circulating immune cells in an age-dependent manner. Therefore, our data imply a CHIP-associated higher susceptibility to a sever course of COVID-19 on the one hand, and under the pressure of SARS-CoV-2 infection, an altered immune regulation in the long run. Based on this study, it is not possible to establish a direct, sole impact on the survival of hospitalized COVID-19 patients. Albeit the question remains to what extent CHIP, whose prevalence increases exponentially with age, indirectly contributes to the risk of death, which also increases depending on age. Our data at least propose that CHIP may be a hidden mechanistic link. Future studies are required to unravel these interconnections as well as the contribution of cardiac injury, as cardiac biomarkers were significantly elevated in the total cohort but not age-dependent and without an impact on survival. Another limitation of our study is the comparatively small number of patients, especially in each age-related subgroup. In view of this, our results need to be validated in further studies including substantially more patients. Finally, the mechanistic link between the different mutations on the one hand and the differently regulated immune response as well as its age dependence, on the other hand, appears worthy of further investigations. Yet, the findings we present here strongly support clonal hematopoiesis being a potent biomarker for early risk stratification and might be used to early guide clinical treatment of patients with COVID-19.

## Data availability statement

The sequence data presented in the study are deposited at the European Genome-phenome Archive (EGA), which is hosted by the EBI and the CRG, under accession number EGAS00001006218: https://ega-archive.org/studies/EGAS00001006218.

## Ethics statement

The studies involving human participants were reviewed and approved by the ethics committees of the Medical Faculties of Heidelberg University, the Ruhr University Bochum, and the University of Münster. The patients/participants provided their written informed consent to participate in this study.

## Author contributions

JS, IH, CM-T, FL, and MW designed the study. KR, BS, JT, HN, AZ, MA, and MW obtained ethics approval. BS, JT, HN, DF, AH, TG, AZ, MF, JZ, JL, UM, MA and MW performed patient recruitment and obtained informed consent. JS, KR, BS, JT, LT, KB, and BK carried out the experiments. MM and TH performed sequencing, bioinformatic analysis and variant interpretation. JS, KR, BS, JT, HN, DF, BK, and TG performed data collection. JS, KR, BS, IH, TR, DF, AH, BK, AZ, JL, MA, FL, and MW performed data interpretation. JS wrote the manuscript. All authors critically revised and drafted the manuscript. All authors read and approved the final manuscript.

## Funding

Parts of this work were supported by grants from the German Research Foundation [KFO342-1, ZA428/18-1, and ZA428/21-1 to AZ] and from the Government of North-Rhine Westphalia [CovidDataNet.NRW to KR, TR, HN, BK, MA].

## Acknowledgments

The authors thank Sabine Stegmaier, Jan Pfister, and Ute Krauser from Heidelberg for their comprehensive and excellent technical support.

## Conflict of interest

Author TH declares part ownership of MLL Munich Leukemia Laboratory. Author MM is employed by MLL.

The remaining authors declare that the research was conducted in the absence of any commercial or financial relationships that could be construed as a potential conflict of interest.

## Publisher’s note

All claims expressed in this article are solely those of the authors and do not necessarily represent those of their affiliated organizations, or those of the publisher, the editors and the reviewers. Any product that may be evaluated in this article, or claim that may be made by its manufacturer, is not guaranteed or endorsed by the publisher.
