# Supplementary material for: Increased prevalence of clonal hematopoiesis of indeterminate potential in hospitalized patients with COVID-19
Source: Front Immunol. 2022 Oct 14;13:968778. doi: 10.3389/fimmu.2022.968778 (PMC9614713; doi:10.3389/fimmu.2022.968778)
Supplement: Supplementary file 1 [file DataSheet_1.pdf]

Supplementary Material

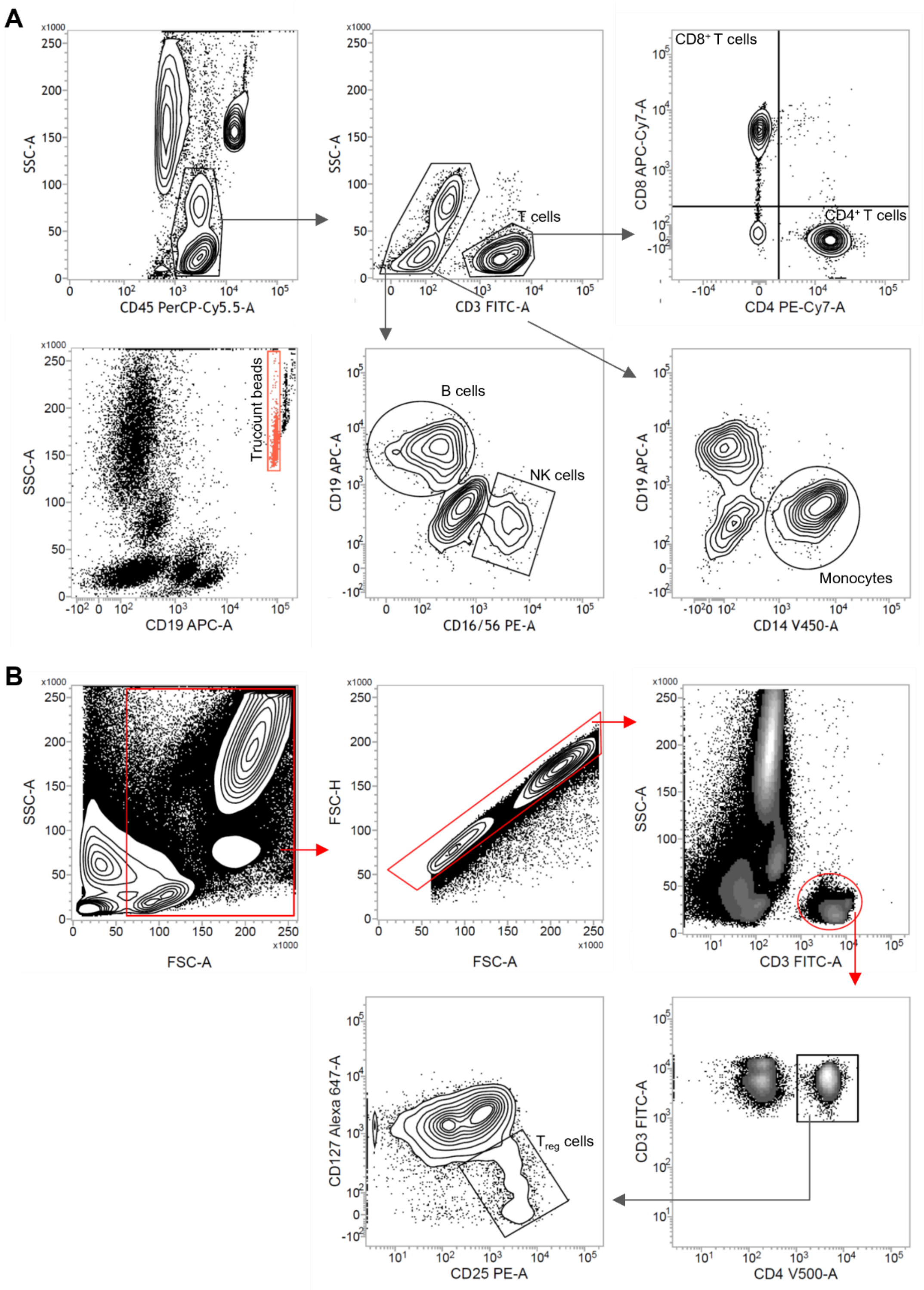

... continued on next page

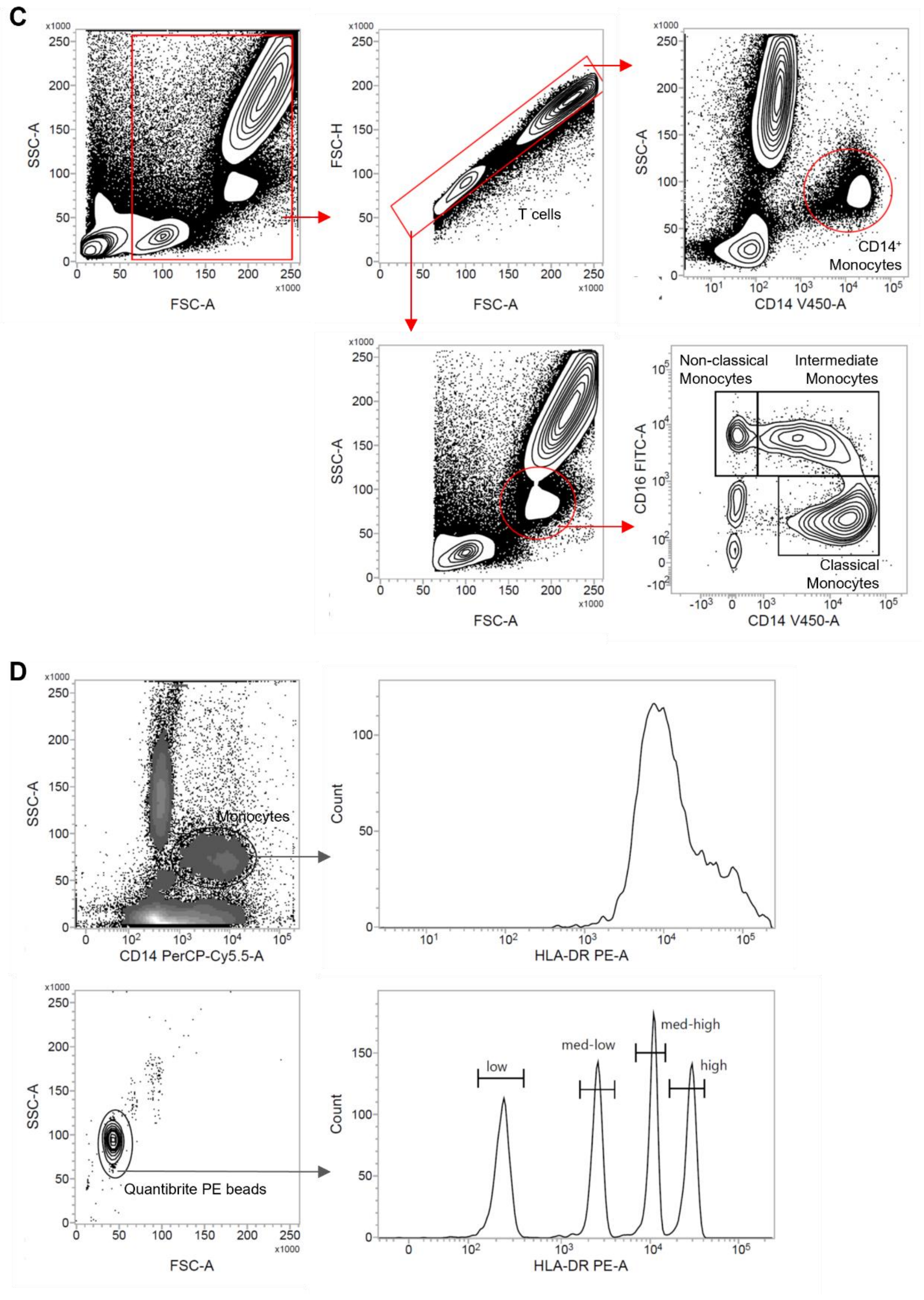

Supplementary Figure 1. Representative gating strategy. (A) Quantification of lymphocyte subsets and monocytes using BD Multitest 6-color TBNK reagent and anti-Human CD14-V450. Trucount beads were used to determine absolute counts. Identification of **(B)** Treg cells and **(C)** monocyte subsets. To calculate the absolute values, the relative results were offset against the results of the TBNK measurement. **(D)** Quantification of HLA-DR expression on monocytes.

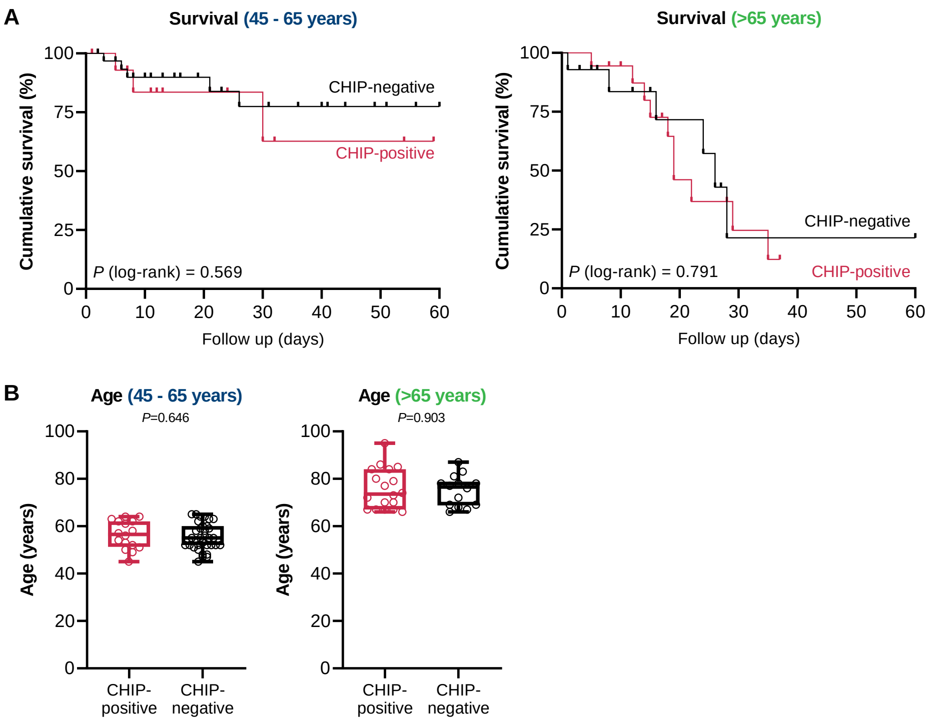

Supplementary Figure 2. Age-adjusted effect of clonal hematopoiesis on patient outcome. (A) Kaplan-Meier survival curves for cumulative survival of CHIP-positive vs. CHIP-negative patients differentiated into two age groups. Statistical evaluations were done using log-rank Mantel-Cox test. **(B)** Age distribution between patients with and without CHIP-driver mutation differentiated for two age groups. Each data point represents an individual patient. Horizontal line within the box marks the median, boxes depict the IQR, and whiskers indicate the total range. Group comparisons were done using two-sided Mann–Whitney U-test. 45-65 years (n=50; CHIP-positive: n=16, CHIP-negative: n=34) and >65 years (n=32; CHIP-positive: n=18, CHIP-negative: n=14).

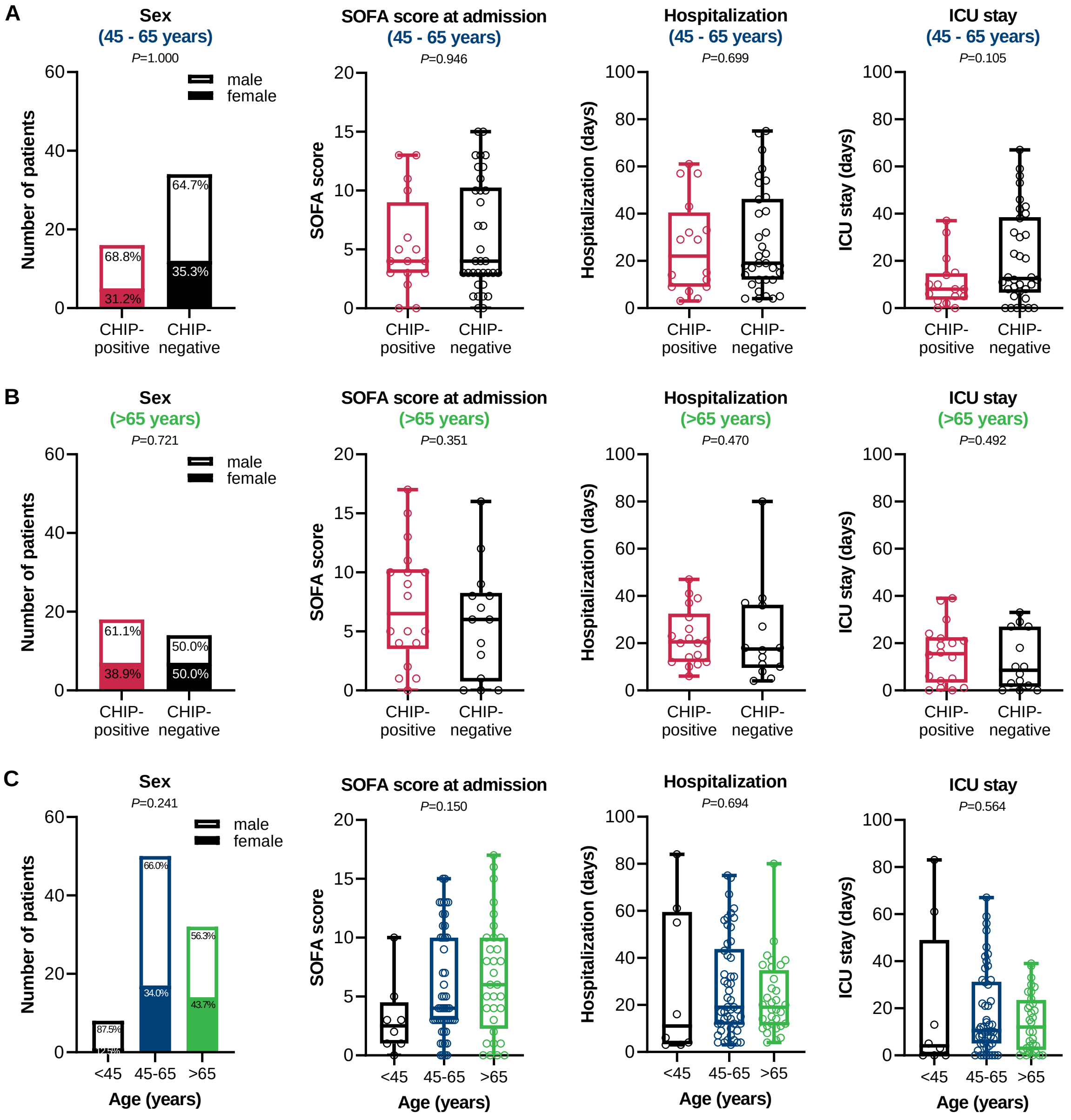

Supplementary Figure 3. Age-adjusted baseline characteristics. Sex, SOFA score at admission, and length of hospitalization and ICU stay for patients aged **(A)** 45 – 65 years (CHIP-positive: n=16; CHIP-negative: n=34) and **(B)** >65 years (CHIP-positive: n=18; CHIP-negative: n=14). Group comparisons were performed by two-sided Mann–Whitney U-test. **(C)** Sex, SOFA score at admission, and length of hospitalization and ICU stay for patients in comparison between the age groups (<45: n=8; 45 – 65: n=50; >65: n=32). Group comparisons were performed using Kruskal-Wallis test. Each data point represents an individual patient. Horizontal line within the box marks the median, boxes depict the IQR, and whiskers indicate the total range.

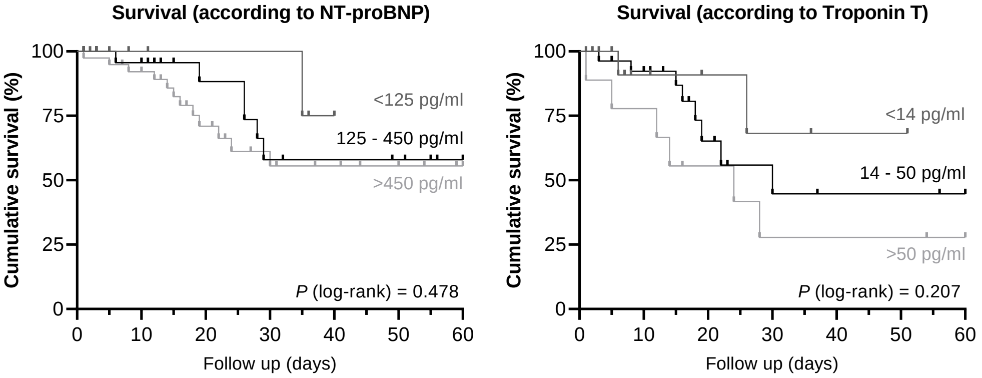

Supplementary Figure 4. Cumulative survival according to cardiac biomarker levels. Kaplan-Meier survival curves for cumulative survival according to NT-proBNP or Troponin T levels at admission. Statistical evaluations were done using log-rank Mantel-Cox test. NT-proBNP <125: n=14, 125 – 450 n=25, >450 n=39; Troponin T <14: n=21, 14 – 50: n=27, >50 n=9.

Supplementary Table 1. CHIP-driver mutations

Patient	Gene	Exon	Chromosomal coordinates	DNA change	Amino acid change	VAF [%]
4	EZH2	10	chr7:148515039	c.1170del	p.Thr391Leufs*33	1.58
4	SF3B1	15	chr2:198266834	c.2098A>G	p.Lys700Glu	1.75
4	SRSF2	1	chr17:74732959	c.284C>A	p.Pro95His	12.1
4	TET2	9	chr4:106190807	c.4085T>C	p.Leu1362Pro	8.5
4	ZRSR2	7	chrX:15827425	c.541T>A	p.Cys181Ser	2.82
8	PPM1D	6	chr17:58740630	c.1535del	p.Asn512Ilefs*2	1.14
10	TET2	5	chr4:106163991	c.3501G>T	p.Arg1167Ser	2.69
12	DNMT3A	15	chr2:25467023	c.1851+1G>A	p.splice site mutation	1.84
13	DNMT3A	18	chr2:25463563	c.2119G>C	p.Gly707Arg	1.37
13	TET2	3	chr4:106156808	c.1709del	p.Pro570Leufs*10	3.68
14	PPM1D	6	chr17:58740630	c.1535del	p.Asn512Ilefs*2	1.13
22	DNMT3A	15	chr2:25467117	c.1758C>G	p.Cys586Trp	1.3
23	DNMT3A	22	chr2:25458593	c.2580G>A	p.Trp860*	1.48
29	TET2	8	chr4:106183006	c.4044+1G>T	p.splice site mutation	12.2
33	SMC1A	19	chrX:53421723	c.2948A>G	p.Tyr983Cys	1.78
33	U2AF1	6	chr21:44514777	c.470A>G	p.Gln157Arg	2.25
36	DNMT3A	7	chr2:25470905	c.855+1G>A	p.splice site mutation	6.2
41	PPM1D	6	chr17:58740535-58740536	c.1439_1440dup	p.Ala481Lysfs*3	2.24
43	PPM1D	6	chr17:58740749	c.1654C>T	p.Arg552*	6.4
45	DNMT3A	23	chr2:25457242	c.2645G>A	p.Arg882His	4.45
47	PPM1D	6	chr17:58740630	c.1535del	p.Asn512Ilefs*2	1.94
49	DNMT3A	7	chr2:25470997-25470998	c.754_763dup	p.Ser255*	2.53
50	CUX1	18	chr7:101844922	c.2378del	p.Pro793Argfs*26	1.12
51	ASXL1	13	chr20:31022233	c.1720-2A>G	p.splice site mutation	11
51	TET2	3	chr4:106156934-106156935	c.1835_1836insG	p.Gly613Trpfs*25	25.5
51	TET2	3	chr4:106156935	c.1836T>G	p.Pro612Pro	29.3
51	TET2	10	chr4:106193761	c.4223del	p.Gly1408Glufs*40	33.4
51	TET2	10	chr4:106193761	c.4223G>A	p.Gly1408Glu	33.3
53	SF3B1	14	chr2:198267486	c.1871T>G	p.Val624Gly	2.18
54	DNMT3A	15	chr2:25467073	c.1802G>A	p.Trp601*	2.91
54	SF3B1	15	chr2:198266834	c.2098A>G	p.Lys700Glu	2.22
55	DNMT3A	7	chr2:25470997-25470998	c.754_763dup	p.Ser255*	1.41
56	DNMT3A	21	chr2:25459837	c.2446C>T	p.Gln816*	2.7
56	DNMT3A	21	chr2:25459806-25459795	c.2477_2478+10del	p.splice site mutation	14
57	DNMT3A	20	chr2:25462006	c.2401A>G	p.Met801Val	23.2
57	PPM1D	6	chr17:58740684-58740685	c.1589dup	p.Pro531Serfs*5	1.56
62	DNMT3A	10	chr2:25469488	c.1279+1G>C	p.splice site mutation	1
64	DNMT3A	18	chr2:25463595	c.2087A>C	p.Gln696Pro	1.56
65	DNMT3A	18	chr2:25463595	c.2087A>C	p.Gln696Pro	2.34
67	SMC3	25	chr10:112361869-112361870	c.3038C>T	p.Ser1013Leu	4.06
68	DNMT3A	14	chr2:25467504	c.1572T>A	p.Cys524*	1.93
71	TET2	7	chr4:106180837	c.3865T>G	p.Cys1289Gly	1.14
71	ZRSR2	5	chrX:15822297	c.376C>T	p.Arg126*	2.49
78	MYD88	2	chr3:38181393	c.406G>T	p.Glu136*	42.8
78	ZRSR2	8	chrX:15833922	c.680del	p.Tyr227Serfs*11	6.5
79	PPM1D	6	chr17:58740546-58740547	c.1450_1451dup	p.Leu484Phefs*2	1.28
80	STAG2	19	chrX:123196964	c.1732-2A>G	p.splice site mutation	2.51
82	DNMT3A	22	chr2:25458609-25458610	c.2559_2563dup	p.Lys855Metfs*28	1.87
89	PPM1D	6	chr17:58740546-58740547	c.1450_1451dup	p.Leu484Phefs*2	1.15

Supplementary Table 2. Infection and organ function-related clinical biomarkers at admission

		CHIP-positive (n=34)	CHIP-negative (n=56)	*P-value*
C-reactive protein, mg/l				0.957
	Median (IQR)	119.3 (65.8 – 185.3)	118.4 (50.7 – 184.4)	
	Range	5.8 – 326.5	15.1 – 457.7	
Procalcitonin, ng/ml				0.458
	Median (IQR)	0.31 (0.15 – 0.58)	0.21 (0.09 – 0.81)	
	Range	0.06 – 7.06	0.05 – 19.91	
		n=33 (97.1%)	n=56 (100%)	
Horovitz index, mmHg				0.224
	Median (IQR)	164.3 (145.9 – 228.5)	155.6 (118.0 – 199.9)	
	Range	60.8 – 348.6	74.0 – 404.0	
		n=28 (83.4%)	n=42 (75.0%)	
Mean arterial pressure, mmHg				0.857
	Median (IQR)	85.0 (79.8 – 96.5)	86.0 (77.0 – 94.5)	
	Range	64.0 – 111.0	63.0 – 124.0	
Catecholamine support				0.665
	No. (%)	15/34 (44.1%)	22/56 (39.3%)	
**NT-proBNP, pg/ml**				**0.018**
	Median (IQR)	991.9 (270.5 – 3726.0)	347.0 (133.8 – 1217.0)	
	Range	79 – 104400	20 – 12592	
		n=29 (85.3%)	n=49 (87.5%)	
**High sensitive Troponin T, pg/ml**				**0.017**
	Median (IQR)	23.0 (16.0 – 44.3)	15.0 (5.3 – 28.5)	
	Range	6.0 – 527.0	2.0 – 131.6	
		n=21 (61.8%)	n=36 (64.3%)	
Creatine kinase, U/l				0.055
	Median (IQR)	68.8 (48.4 – 374.4)	164.3 (66.8 – 679.6)	
	Range	12.1 – 954.9	10.0 – 3455	
		n=28 (82.4%)	n=38 (67.9%)	
D-dimer, mg/l				0.558
	Median (IQR)	1.66 (0.89 – 3.78)	1.46 (0.69 – 5.09)	
	Range	0.40 – 35.00	0.30 – 35.00	
		n=29 (85.3%)	n=52 (92.9%)	
Lactate dehydrogenase, U/l				0.354
	Median (IQR)	455.5 (325.3 – 555.9)	453.0 (367.0 – 652.8)	
	Range	191.0 – 882.0	187.0 – 652.8	
Serum creatinine, mg/dl				0.111
	Median (IQR)	1.02 (0.75 – 1.66)	0.84 (0.70 – 1.17)	
	Range	0.44 – 8.65	0.33 – 10.81	
		n=32 (94.1%)	n=55 (98.2%)	
Renal replacement therapy				0.273
	No. (%)	8/34 (23.5%)	8/56 (14.3%)	

P-values from two-sided Fisher's exact test for categorical variables and from two-sided Mann–Whitney U-test for comparison. Statistically significant results ($P \leq 0.05$) are highlighted by bold print.

Supplementary Table 3. Immune parameters at admission

		CHIP-positive (n=12)	CHIP-negative (n=30)	*P-value*
T cells, cells/µL				0.731
	Median (IQR)	515.3 (393.0 – 729.0)	582.3 (340.0 – 872.9)	
	Range	272.6 – 1027.0	180.2 – 1624.0	
$CD4^+$ T cells, cells/µL				0.554
	Median (IQR)	297.4 (200.3 – 392.9)	291.7 (210.0 – 518.7)	
	Range	188.5 – 691.9	131.0 – 1247.0	
$CD8^+$ T cells, cells/µL				0.967
	Median (IQR)	187.2 (140.6 – 268.1)	183.4 (100.5 – 315.2)	
	Range	64.4 – 372.1	27.8 – 605.5	
T_{reg} cells, cells/µL				0.928
	Median (IQR)	26.8 (20.9 – 49.0)	27.6 (18.2 – 40.0)	
	Range	10.6 – 74.2	9.3 – 81.9	
NK cells, cells/µL				0.924
	Median (IQR)	166.0 (85.0 – 285.9)	162.2 (100.9 – 244.4)	
	Range	27.9 – 1116	52.0 – 1225	
B cells, cells/µL				0.500
	Median (IQR)	177.7 (107.6 – 319.7)	155.1 (90.5 – 246.3)	
	Range	57.2 – 459.7	32.0 – 674.8	
Monocytes, cells/µL				0.837
	Median (IQR)	267.6 (180.5 – 569.5)	275.1 (185.5 – 370.4)	
	Range	71.1 – 1824.0	28.4 – 1098.0	
Classical monocytes, cells/µL				0.794
	Median (IQR)	244.0 (149.3 – 499.9)	235.2 (151.9 – 353.7)	
	Range	56.6 – 917.1	13.13 – 846.5	
Intermediate monocytes, cells/µL				0.989
	Median (IQR)	5.3 (2.3 – 13.4)	4.7 (2.9 – 17.4)	
	Range	0.5 – 45.0	0.0 – 79.0	
Non-classical monocytes, cells/µL				0.650
	Median (IQR)	5.5 (3.0 – 12.7)	9.2 (2.5 – 19.9)	
	Range	0.5 – 346.1	0.3 – 230.3	
HLA-DR on monocytes, molecules/cell				0.837
	Median (IQR)	18999 (13754 – 21092)	16833 (10162 – 26755)	
	Range	7272 – 37283	6755 – 41432	
Plasma IFN-γ, pg/mL				0.289
	Median (IQR)	7.67 (3.34 – 17.60)	3.29 (1.66 – 8.85)	
	Range	0.52 – 35.63	0.34 – 30.29	
	Missing data: value bellow LOD, no. (%)	6 (50%)	9 (30%)	
Plasma IP-10, pg/mL				0.611
	Median (IQR)	700.4 (305.7 – 1400.0)	646.1 (316.2 – 1167.0)	
	Range	84.9 – 2224.0	62.4 – 5011.0	
Plasma TGF-β, pg/mL				0.417
	Median (IQR)	1315.0 (1070.0 – 1515.0)	1116.0 (825.8 – 1701.0)	
	Range	927.5 – 1577.0	589.6 – 2411.0	
Plasma IL-6, pg/mL				0.814
	Median (IQR)	31.8 (22.0 – 239.5)	49.5 (24.3 – 72.8)	
	Range	21.8 – 308.0	22.0 – 456.6	
	Missing data: value bellow LOD, No. (%)	7 (41.7%)	15 (50.0%)	

IFN-γ response to αCD3/αCD28 stimulation, pg/mL			0.064
Median (IQR)	3.06 (1.18 – 7.19)	35.61	
Range	0.41 – 9.72	0.32 – 1407.00	
Missing data:			
value bellow LOD, no. (%)	1 (8.3%)	6 (20.0%)	
not analyzed, no. (%)	0	1 (3.3%)	
IP-10 response to αCD3/αCD28 stimulation, pg/mL			0.506
Median (IQR)	265.8 (156.9 – 352.9)	366.4 (81.5 – 674.1)	
Range	103.2 – 469.8	31.2 – 1354.0	
Missing data:			
not analyzed, no. (%)	0	1 (3.3%)	

P-values from two-sided Mann–Whitney U-test.

Supplementary Table 4. Differential blood count parameters

		CHIP-positive (n=34)	CHIP-negative (n=56)	*P-value*
Erythrocytes at admission, cells/pL				0.279
	Median (IQR)	4.2 (3.4 – 4.7)	4.4 (3.8 – 4.8)	
	Range	2.3 – 5.4	1.9 – 5.8	
		n=34	n=54	
Erythrocytes at discharge, cells/pL				0.903
	Median (IQR)	3.3 (3.0 – 4.4)	3.5 (3.0 – 4.1)	
	Range	2.3 – 5.1	2.4 – 5.7	
		n=34	n=56	
Erythrocytes at day 7, cells/pl				0.618
	Median (IQR)	3.3 (2.8 – 4.2)	3.5 (3.1 – 4.0)	
	Range	2.5 – 5.2	2.6 – 4.9	
		n=24	n=40	
Erythrocytes at day 14, cells/pL				0.739
	Median (IQR)	3.2 (2.7 – 3.4)	3.0 (2.8 – 3.5)	
	Range	2.5 – 4.0	2.4 – 4.4	
		n=13	n=30	
Thrombocytes at admission, cells/nL				0.320
	Median (IQR)	222.0 (180.3 – 274.0)	200.5 (141.3 – 284.3)	
	Range	70.0 – 813.0	69.0 – 773.0	
		n=34	n=54	
Thrombocytes at discharge, cells/nL				0.362
	Median (IQR)	262.0 (137.8 – 383.8)	273.5 (208.8 – 378.8)	
	Range	14.8 – 565.0	94.0 – 580.0	
		n=34	n=54	
Thrombocytes at day 7, cells/nl				0.627
	Median (IQR)	285.5 (220.5 – 362.8)	260.5 (203.3 – 378.0)	
	Range	49.0 – 556.0	58.0 – 516.0	
		n=24	n=40	
Thrombocytes at day 14, cells/nL				0.186
	Median (IQR)	200.0 (125.5 – 254.5)	228.5 (198.0 – 321.8)	
	Range	68.0 – 420.0	95.0 – 478.0	
		n=13	n=30	
Leucocytes at admission, cells/nL				0.079
	Median (IQR)	9.7 (6.0 – 12.9)	7.5 (4.9 – 11.6)	
	Range	3.5 – 41.1	1.7 – 16.3	
		n=34	n=54	
Leucocytes at discharge, cells/nL				0.097
	Median (IQR)	10.9 (7.8 – 15.0)	9.2 (5.5 – 13.3)	
	Range	3.8 – 27.7	3.2 – 32.7	
		n=34	n=56	
Leucocytes at day 7, cells/nl				0.594
	Median (IQR)	11.9 (10.0 – 15.3)	10.9 (8.1 – 16.4)	
	Range	6.0 – 25.8	4.6 – 29.4	
		n=24	n=40	
**Leucocytes at day 14, cells/nL**				**0.024**
	Median (IQR)	16.9 (14.0 – 23.5)	10.1 (7.6 – 14.5)	
	Range	4.2 – 34.7	4.4 – 31.5	
		n=13	n=30	
Lymphocytes at admission, cells/nL				0.907
	Median (IQR)	0.8 (0.4 – 1.3)	0.8 (0.6 – 1.0)	
	Range	0.2 – 2.3	0.0 – 10.4	

		n=33	n=53	
**Lymphocytes at discharge, cells/nL**				**0.002**
	Median (IQR)	1.1 (0.6 – 1.4)	1.6 (1.1 – 2.0)	
	Range	0.2 – 3.3	0.3 – 5.3	
		n=34	n=53	
Lymphocytes at day 7, cells/nl				0.941
	Median (IQR)	1.0 (0.7 – 1.5)	1.0 (0.7 – 1.5)	
	Range	0.3 – 3.4	0.1 – 3.6	
		n=24	n=39	
Lymphocytes at day 14, cells/nL				0.214
	Median (IQR)	1.1 (0.6 – 1.5)	1.4 (0.8 – 2.0)	
	Range	0.3 – 3.6	0.2 – 5.0	
		n=13	n=30	
Monocytes at admission, cells/nL				0.410
	Median (IQR)	0.4 (0.2 – 0.7)	0.4 (0.2 – 0.5)	
	Range	0.1 – 2.4	0.0 – 7.0	
		n=33	n=53	
Monocytes at discharge, cells/nL				0.787
	Median (IQR)	0.6 (0.3 – 0.9)	0.5 (0.3 – 0.9)	
	Range	0.1 – 4.7	0.1 – 2.4	
		n=34	n=53	
Monocytes at day 7, cells/nl				0.329
	Median (IQR)	0.6 (0.4 – 0.9)	0.6 (0.4 – 0.8)	
	Range	0.0 – 1.7	0.1 – 2.4	
		n=24	n=39	
Monocytes at day 14, cells/nL				0.268
	Median (IQR)	0.8 (0.5 – 1.2)	0.5 (0.4 – 1.0)	
	Range	0.1 – 4.7	0.1 – 2.4	
		n=13	n=30	
Neutrophils at admission, cells/nL				0.136
	Median (IQR)	7.3 (4.9 – 10.6)	6.5 (3.9 – 9.5)	
	Range	2.8 – 37.5	0.4 – 82.4	
		n=33	n=53	
**Neutrophils at discharge, cells/nL**				**0.024**
	Median (IQR)	8.0 (5.4 – 13.2)	5.5 (3.6 – 9.8)	
	Range	1.0 – 23.1	1.6 – 24.7	
		n=34	n=53	
Neutrophils at day 7, cells/nl				0.273
	Median (IQR)	10.4 (8.0 – 13.8)	8.6 (5.9 – 14.1)	
	Range	4.7 – 26.2	2.9 – 27.4	
		n=24	n=38	
**Neutrophils at day 14, cells/nL**				**0.011**
	Median (IQR)	16.6 (11.4 – 22.9)	7.6 (5.2 – 13.7)	
	Range	3.1 – 33.8	2.9 – 22.1	
		n=13	n=30	
Eosinophils at admission, cells/nL				0.919
	Median (IQR)	0.01 (0.00 – 0.03)	0.01 (0.00 – 0.03)	
	Range	0.00 – 0.44	0.00 – 0.56	
		n=33	n=53	
**Eosinophils at discharge, cells/nL**				**0.034**
	Median (IQR)	0.04 (0.00 – 0.20)	0.10 (0.03 – 0.34)	
	Range	0.00 – 1.18	0.00 – 3.26	
		n=34	n=53	

Eosinophils at day 7, cells/nl				0.601
	Median (IQR)	0.08 (0.01 – 0.12)	0.05 (0.01 – 0.11)	
	Range	0.00 – 0.91	0.00 – 0.45	
		n=24	n=38	
Eosinophils at day 14, cells/nL				0.348
	Median (IQR)	0.07 (0.01 – 0.34)	0.11 (0.06 – 0.24)	
	Range	0.00 – 0.63	0.00 – 1.24	
		n=13	n=30	
Basophils at admission, cells/nL				0.594
	Median (IQR)	0.02 (0.01 – 0.03)	0.02 (0.01 – 0.03)	
	Range	0.00 – 0.11	0.00 – 0.14	
		n=33	n=53	
Basophils at discharge, cells/nL				0.231
	Median (IQR)	0.03 (0.01 – 0.05)	0.04 (0.02 – 0.07)	
	Range	0.00 – 0.18	0.00 – 0.40	
		n=33	n=53	
Basophils at day 7, cells/nl				0.299
	Median (IQR)	0.04 (0.01 – 0.07)	0.03 (0.01 – 0.06)	
	Range	0.00 – 0.35	0.00 – 0.20	
		n=24	n=39	
Basophils at day 14, cells/nL				0.473
	Median (IQR)	0.05 (0.02 – 0.10)	0.04 (0.02 – 0.07)	
	Range	0.00 – 0.14	0.00 – 0.20	
		n=13	n=30	

P-values from two-sided Mann–Whitney U-test. Statistically significant results ($P \leq 0.05$) are highlighted by bold print.
